# Exhaled volatile substances in children suffering from type 1 diabetes mellitus: results from a cross-sectional study

**DOI:** 10.1038/s41598-019-52165-x

**Published:** 2019-10-31

**Authors:** Phillip Trefz, Juliane Obermeier, Ruth Lehbrink, Jochen K. Schubert, Wolfram Miekisch, Dagmar-Christiane Fischer

**Affiliations:** 1 Department of Anesthesiology and Intensive Care Medicine, Rostock Medical Breath Research Analytics and Technologies (ROMBAT), Rostock University Medical Centre, Rostock, Germany; 2 Department of Pediatrics, Rostock University Medical Centre, Rostock, Germany

**Keywords:** Diagnostic markers, Type 1 diabetes

## Abstract

Monitoring metabolic adaptation to type 1 diabetes mellitus in children is challenging. Analysis of volatile organic compounds (VOCs) in exhaled breath is non-invasive and appears as a promising tool. However, data on breath VOC profiles in pediatric patients are limited. We conducted a cross-sectional study and applied quantitative analysis of exhaled VOCs in children suffering from type 1 diabetes mellitus (T1DM) (n = 53) and healthy controls (n = 60). Both groups were matched for sex and age. For breath gas analysis, a very sensitive direct mass spectrometric technique (PTR-TOF) was applied. The duration of disease, the mode of insulin application (continuous subcutaneous insulin infusion vs. multiple daily insulin injection) and long-term metabolic control were considered as classifiers in patients. The concentration of exhaled VOCs differed between T1DM patients and healthy children. In particular, T1DM patients exhaled significantly higher amounts of ethanol, isopropanol, dimethylsulfid, isoprene and pentanal compared to healthy controls (171, 1223, 19.6, 112 and 13.5 ppbV vs. 82.4, 784, 11.3, 49.6, and 5.30 ppbV). The most remarkable differences in concentrations were found in patients with poor metabolic control, i.e. those with a mean HbA_1c_ above 8%. In conclusion, non-invasive breath testing may support the discovery of basic metabolic mechanisms and adaptation early in the progress of T1DM.

## Introduction

Type 1 diabetes mellitus (T1DM) is a chronic metabolic disease with about 86,000 newly diagnosed children worldwide every year^1–3^. Due to the autoimmune destruction of pancreatic ß-cells with a concomitantly decreasing and finally completely missing insulin secretion^4^, patients rely on lifelong administration of insulin. This can either be done via continuous subcutaneous infusion (CSII, pump) or multiple daily injections (MDI). However, self-monitoring of glucose for adjustment of insulin dosages and carbohydrate intake is required to control for hypo- and hyperglycemia. While hypoglycemia can cause seizures, coma or even death, hyperglycemia and/or glycemic variability are potent triggers of secondary organ damage (e.g. micro-and macrovasculopathy leading to cardiovascular disease, diabetic nephropathy, neuropathy and retinopathy) via the induction of oxidative stress^5–14^. Furthermore, complications progress silent, become symptomatic usually after years and are mostly irreversible at the time of diagnosis. In T1DM patients the duration of disease can approximate biological age, i.e. patients are at risk to suffer from diabetes related comorbidities already at young adult age^2,15,16^. For this reason, long-term metabolic control is of great relevance and measures to maintain or even increase adherence to unpleasant metabolic control via an immediate feedback, rather than pointing to an invisible risk in the future are especially important.

Although glycemic variability can be assessed via continuous glucose monitoring and/or estimated from self-monitored blood glucose data, the amount of HbA_1c_ is an established marker of long-term glycemic control and a mean HbA_1c_ below 7.5% is judged as an indicator of adequate metabolic control^17–19^. By contrast, the transient release of metabolites related to fluctuating levels of oxidative stress are hard to monitor *in-vivo*.

The analysis of volatile organic compounds (VOCs) in exhaled breath of patients may offer a solution to this problem. Breath biomarkers could provide new and unique insights into metabolic, physiologic or pathophysiological processes. As VOCs are exhaled shortly after their production, they may deliver information quicker than invasive techniques and enable insights into short-term variations of metabolites. Apart from the typical sweet smell of breath in patients with severe diabetic ketoacidosis, hundreds of potentially endogenous volatile metabolites have been quantified in trace amounts (~ppbV-pptV range) in human exhalation under physiological and pathophysiological conditions^20–27^. Exhaled amounts of monomethylated alkanes and C_4_–C_20_ alkanes have been suggested as markers of oxidative stress in diabetic adults^28^. Several pilot studies investigated the exhaled amounts of acetone, isoprene and a variety of other VOCs as surrogate markers of blood glucose and/or as diagnostic tool for detection of hyper- or hypoglycemic events under well-defined experimental settings, i.e. intraveneous or oral glucose challenge, hypo- or euglycemic clamps^29–34^. While an association between metabolic state and certain VOCs could be detected under such circumstances, a single measurement of either acetone or isoprene failed to reflect blood glucose in pediatric T1DM patients^35^. Instead, a positive association between blood ketones and breath acetone was noted.

While breath gas analysis is certainly not suitable to assess the glycemic state, it might help to gain knowledge on metabolic adaption under conditions of real-life, i.e. simultaneous investigation of a broad panel of VOCs (“volatilome”) in real-time. This can e.g. be done with proton-transfer-reaction time-of-flight mass spectrometry (PTR-ToF-MS). Very recently, we applied this technique in children with mild-to-moderate chronic kidney disease and healthy controls^36^. Within the frame of this study, we also invited children suffering from T1DM that were treated at our institution in order to examine volatile metabolites in the breath of pediatric T1DM patients and healthy controls.

## Methods

### Patients and controls

The study received appropriate ethics committee approval from the institutional Ethics Committee (University Medical Centre Rostock, Rostock, Germany) in accordance with the Declaration of Helsinki (approval number: A 2012 0103). All subjects and/or their parents gave written and informed consent for their participation in the study.

*Inclusion criteria*: age 4–18 years, C-peptide below 0.3 nmol/l and either multiple daily insulin injections (MDII) or continuous subcutaneous insulin infusions (CSII, pump therapy).

Both, healthy controls and T1DM patients were excluded in any case of febrile illness during the last three months, chronic inflammatory-/rheumatic disease (e.g. Crohn’s disease, rheumatoid arthritis), hepatitis, HIV, glucocorticoid treatment, liver-, renal-, or cardiac failure, or hereditary dyslipidaemia.

### Study investigations

All patients were seen in the afternoon and breath sampling was done after a routine visit in our outpatient clinic. Demographic and clinical data including the history of disease, daily insulin dosage related to body weight as well as results from routine laboratory analysis were gathered by interview and chart review, respectively. The mean HbA_1c_ during the last 12 months was considered as surrogate marker of long-term metabolic control and a mean HbA_1c_ above 8% was considered as poor.

A trained physician measured weight and height via electronic scales and a fixed stadiometer. Blood pressure (BP) was measured according to the updated Task Force Report on high blood pressure by using an oscillometric device (Dinamap 1846SX; Critikon, Tampa, USA). Calculations of individual age- and sex-related standard deviation scores (SD scores) for height, weight, BMI and BP were done as previously described^7,37–39^. Patients were classified as hypertensive in case of BP values above the height- and sex-related 95th percentile.

### Breath sampling and analysis

Breath was sampled continuously during a five minute period and VOCs were analysed in real time by high-resolution proton-transfer-reaction-time-of-flight- mass-spectrometery (PTR-ToF-MS). For this purpose, a PTR-ToF-MS 8000 (Ionicon Analytik GmbH, Innsbruck, Austria) was used essentially as described before^36,40,41^. All participants breathed evenly through the mouth into a sterile T-piece that did not introduce a breathing resistance. The T-piece enabled sampling in side-stream mode of exhaled and inhaled breath (sampling flow rate was 20 ml/min) into an inert and heated (75 °C) transfer line (silcosteel, ID 0.75 mm, Restek, Bellafonte, USA). The transfer line was connected to the drift tube (75 °C, 610 V, 2.3 mbar) of the PTR-ToF-MS. Within the drift tube, the proton transfer reaction between VOCs and H_3_O^+^ occured prior to transfer of the protonated VOCs into the high resolution reflectron time-of-flight mass spectrometer (Tofwerk AG, Thun, Switzerland). The mass and time resolution were ~4000 m/Δm and 200 ms, respectively. The mass scale was recalibrated every minute using the H_3_O^+^-isotope (21.023), NO^+^ (29.998) and C_3_H_6_O (59.049). For quantification of individual VOCs, the intensity measured in counts per seconds (cps) during the third minute of the sampling interval was averaged and normalized onto primary (H_3_O^+^) ion count. A liquid calibration unit (constant total gas flow 1000 ml min^−1^, water flow 0.05 ml min^−1^, 75 °C) was used for external calibration of isoprene, isopropanol, C_1_–C_10_ aldehydes, acetone, ethanol and dimethylsulfide^36^.

### Data processing

A custom made Matlab-based data processing algorithm (“breath tracker”, Matlab version 7.12.0.635, R2011a) enabled the automatic recognition of expiratory and inspiratory phases. Acetone was used as tracker mass^36,40,41^.

Seven VOCs that are either related to metabolic or physiologic processes relevant for T1DM (e.g. glucose metabolism) or have been proposed in the literature as potential markers for metabolic processes and thus may be relevant for diabetes, were selected for further data analysis. VOCs from healthy controls and T1DM patients were tested for differences related to anthropometric and clinical characteristics. In addition, we investigated our findings relative to the mode of insulin therapy (MDII vs. CSII) and the mean HbA_1c_, i.e. a mean HbA_1c_ below and above 8% was used to discriminate between good and poor long-term metabolic control.

### Statistics

For statistical analysis and visualization of results SPSS statistical package 22 (SPSS Inc. Chicago, Illinois, USA), and Sigma Plot Version 10 (Jandel Scientific Inc.) were used. Normal distribution of data was evaluated by the Kolmogorov-Smirnov test and comparison between groups was done using Student’s t-test or Mann-Whitney U test, if appropriate. For computation of correlations, Spearman’s rho test was used. All p-values were two-sided and a p-value below 0.05 was considered significant. Data are given as mean ± standard deviation (sd) or median and range, where appropriate.

## Results

### Characteristics of patients and controls

**Figure 1:**
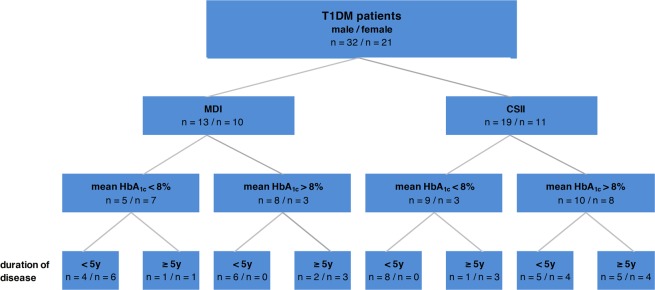
Distribution of patients according to sex, mode of therapy, long-term metabolic control and duration of disease.

A total of 53 T1DM patients (32 males, 21 females) was enrolled in parallel to 60 healthy controls (28 males, 32 females). All patients were in a stable metabolic state and breath gas analysis was done adjacent to a regular consultation in our outpatient clinic. The categorization of patients according to sex, mode of therapy (MDI vs. CSII), long-term metabolic control (mean HbA1c below or above 8%) and duration of disease (less than 5 years vs 5 years or longer) is presented in Fig. 1. Standardized anthropometric data and clinical characteristics of patients and controls are given in Table 1. None of these parameters were related to sex, duration of disease, or mode of therapy. However, patients with poor long-term metabolic control showed significantly higher cholesterol (4.76 ± 1.02 mmol/l vs 3.95 ± 0.74 mmol/l; p < 0.001), LDL-cholesterol (2.73 ± 0.88 mmol/l vs 2.03 ± 0,60 mmol/l; p < 0.001), triglycerides (1.47 ± 1.22 mmol/l vs 0.78. ± 0.52 mmol/l; p < 0.001) and higher blood pressure (standardized BP_sys_: 2.28 ± 1.07 vs 1.13 ± 0.96; p < 0.001; standardized BP_dias_: 1.35 ± 1.34 vs 0.83 ± 0.97; p < 0.001) (Supplement 1).

**Table 1:** Anthropometric and clinical characteristics of patients and controls.

	Patients (32 m/21 f)	Controls (28 m/32 f)	P
Age [year]	12.36 ± 3.15*	13.67 ± 2.80*	0.024
height [SDS]	−0.05 ± 0.87**	0.50 ± 1.06**	0.004
weight [SDS]	0.23 ± 0.78	0.28 ± 0.91	0.747
BMI [SDS]	0.35 ± 0.69*	0.06 ± 0.82*	0.051
BP_sys_ [SDS]	1.76 ± 1.17**	1.07 ± 1.24**	0.003
BP_dias_ [SDS]	1.11 ± 1.21**	0.12 ± 1.30**	<0.001
Duration of Disease [year]	4.75 (0.17–15.25)		
Glucose [mmol/l]	9.78 ± 4.67		
HbA1c [%]			
at time of examination	8.60 ± 1.58		
mean during last year	8.59 ± 1.47		
Cholesterol [mmol/l]	4.39 ± 0.98		
LDL-Cholesterol [mmol/l]	2.40 ± 0.84		
HDL-Cholesterol [mmol/l]	1.63 ± 0.34		
Triglyceride [mmol/l]	1.16 ± 1.01		
normalized Insulindosage [IE/kg/d]	0.37 (0.19–1.05)		

Superscripts denote significant differences between patients and controls (*p < 0.05; **p < 0.01).

### VOC analysis and breath profiles

**Figure 2:**
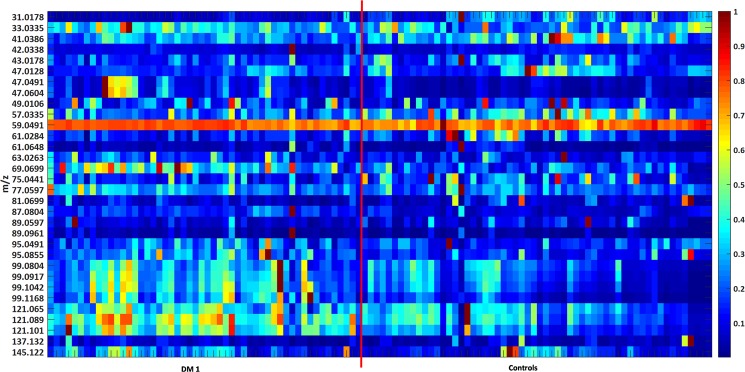
Heatmap based on normalized data of 33 mass traces (31 to 145 m/z) in breath of T1DM patients (left) and healthy controls (right). Data was normalized onto maximum concentration for emphasis of relative differences. Red color represents relatively high concentrations, blue color represents relatively low concentrations.

More than 300 VOCs were detectable in the breath of pediatric T1DM patients and healthy controls. Only VOCs with expiratory concentrations higher than inspiratory concentrations were considered in detail. Figure 2 shows a heatmap of 33 potential VOCs that fulfilled these criteria.

Out of these 33 VOCs, we focused on seven volatile metabolites with a known or postulated relation to metabolic processes which could be relevant within patients suffering from T1DM.

In particular, ethanol (47.049 m/z), acetone (59.049 m/z), isopropanol (61.064 m/z), dimethyl sulfide (DMS; 63.026 m/z), isoprene (69.069 m/z), pentanal (87.080 m/z), and limonene (137.132 m/z) were assessed in detail.

**Figure 3:**
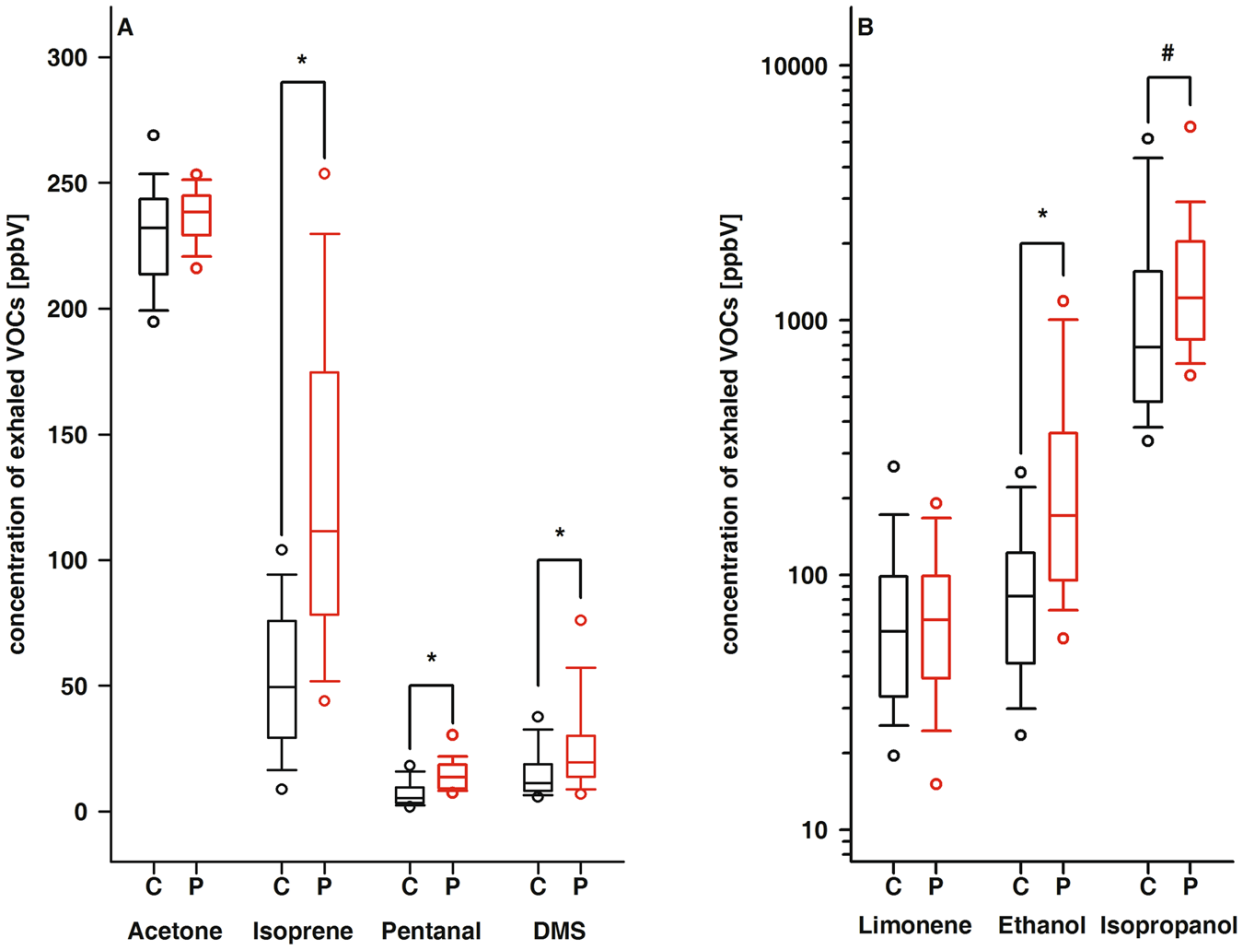
Box plots of exhaled concentrations of acetone, isoprene, pentanal and DMS (**A**) as well as limonene, ethanol and isopropanol (**B**). Black box plots: healthy controls; red box plots: T1DM patients; * and ^#^ indicate statistically significant differences with p < 0.001 and p = 0.002, respectively.

While noticable differences between controls and patients were observed for ethanol, isopropanol, DMS, isoprene, and pentanal, comparable concentrations of acetone and limonene were exhaled by both groups (Table 2 and Fig. 3). Further, we discriminated patients according to long-term metabolic control, i.e. a mean HbA_1c_ below and above 8%. Compared to healthy controls, either cohort of T1DM patients exhaled significantly higher amounts of ethanol, DMS, isoprene, and pentanal. Additionally, the alveolar concentrations of isopropanol and pentanal were significantly different in patients with adequate and poor long-term metabolic control.

**Table 2:** Exhaled amounts of selected alveolar VOCs in the study population.

	Ethanol [ppbV]	Acetone [ppbV]	Isopropanol [ppbV]	DMS [ppbV]	Isoprene [ppbV]	Pentanal [ppbV]	Limonen [ppbV]
Controls	82.4^a,b,c^ (20.7–554)	232* (186–306)	784^a,b^ (287–28,963)	10.0^a,b,c^ (0.99–151)	49.6^a,b,c^ (7.44–153)	5.30^a,b,c^ (1.36–36.9)	51.8 (4.81–1,192)
Patients	171^a^ (44.7–1856)	238* (204–256)	1223^a^ (481–15011)	19.6^a^ (6.49–77.2)	112^a^ (8.36–291)	13.5^a^ (6.11–101)	66.7 (13.9–513)
HbA1c < 8%	150^b^ (71.6–1203)	238 (211–256)	924^c^ (481–2509)	20.0^b^ (4.53–89.6)	105^b^ (49.0–291)	10.6^b,d^ (6.11–30.9)	66.7 (14.8–513)
HbA1c > 8%	221^c^ (44.7–1856)	240 (204–255)	1607^b,c^ (686–15011)	17.1^c^ (4.28–44.1)	118^c^ (8.36–265)	16.2^c,d^ (8.27–101)	66.8 (13.9–209)

Data is given as median and range. Superscripts denote significantly different concentrations of the respective analytes between identically labelled groups. DMS, dimethylsulfide

^a^controls vs patients: p < 0.001 for ethanol, DMS, isoprene, and pentanal, p = 0.002 for isopropanol; ^b^controls vs patients with good metabolic control (HbA_1c_ < 8%): p < 0.001 for ethanol, DMS, isoprene, and pentanal; ^c^controls vs patients with poor metabolic control (HbA_1c_ > 8%): p < 0.001 for ethanol, isopropanol, isoprene, and pentanal, p = 0.03 for DMS; ^d^patiens with poor vs those with good metabolic control: p < 0.001 for isopropanol and p = 0.012 for pentanal.

**Figure 4:**
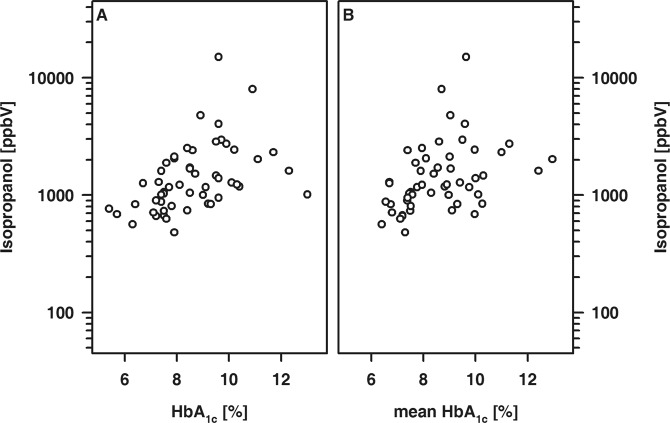
Association between exhaled amounts of isopropanol and HBA_1c_ determined either at time of examination (**A**; R = 0.57, p < 0.001) or calculated as mean of the last 12 months prior to examination (**B**; R = 0.49; p < 0.001).

**Figure 5:**
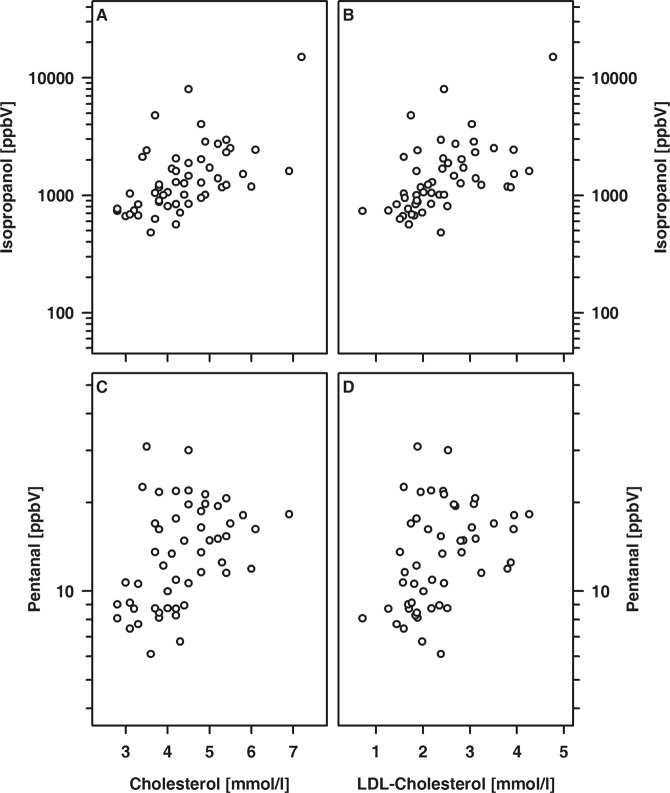
Association between cholesterol (**A,C**) and either isopropanol (**A**) or pentanal (**C**) as well as between LDL-cholesterol (**B,D**) and either isopropanol (**B**) or pentanal (**D**). (**A**) R = 0.58; (**B**) R = 0.60; (**C**) R = 0.48; (**D**) R = 0.46; each p ≤ 0.001.

In T1DM patients, the exhaled amount of isopropanol was positively related to the HbA_1c_ at the time of examination and the mean HbA_1c_ (Fig. 4). Furthermore, the exhaled amounts of isopropanol and pentanal were associated and either VOC showed an association with the cholesterol and LDL-cholesterol concentration, respectively (Fig. 5).

**Figure 6:**
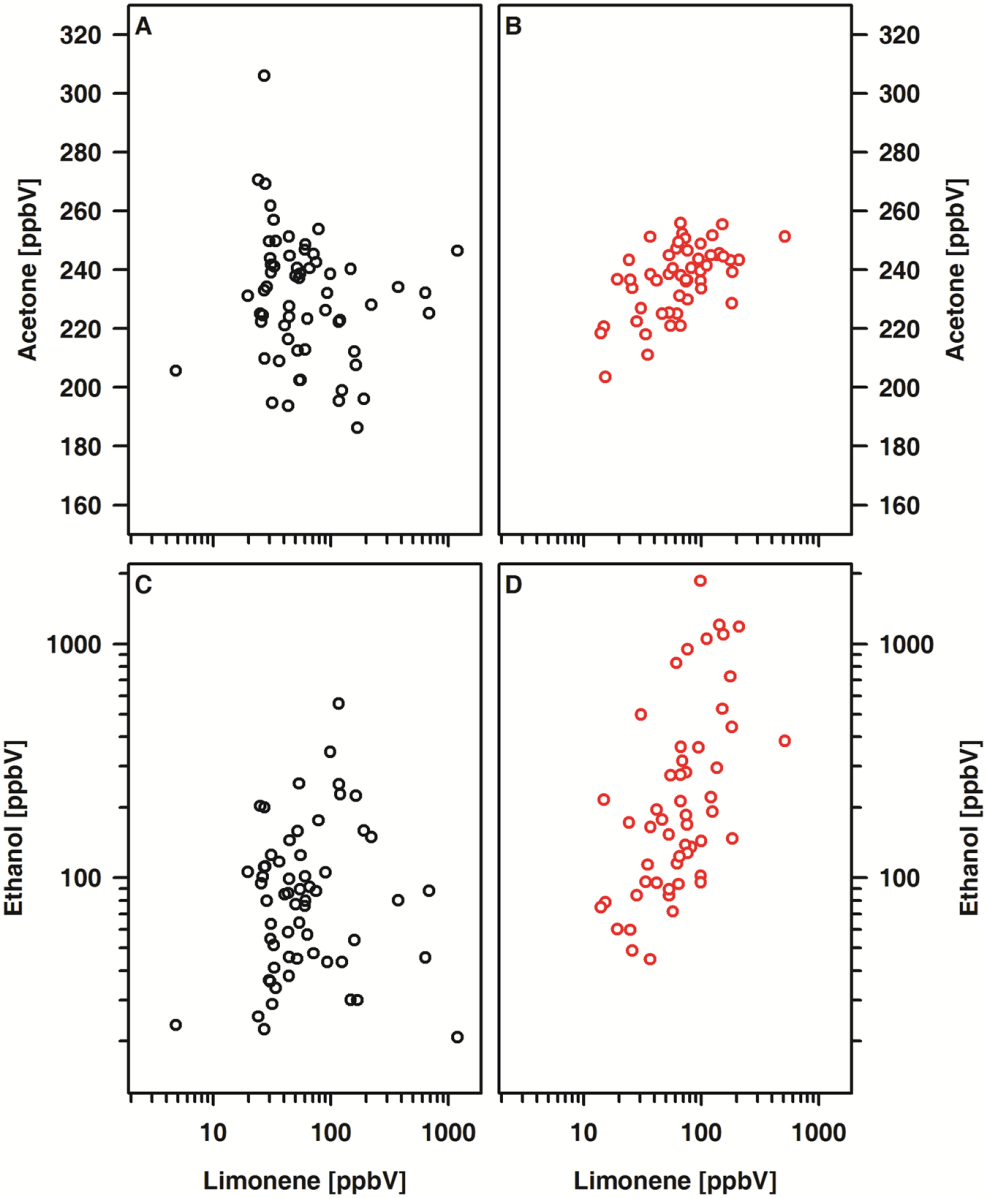
Association between acetone and either limonene (**A,B**) or ethanol (**C,D**) in patients (**A,C**) and controls (**B,D**). (**A**) R = 0.51; (**C**) R = 0.61, each p < 0.001.

Within the patient cohort, positive relations between acetone and limonene (r = 0.51, p < 0.001) as well as between acetone and ethanol (r = 0.61, p < 0.001) were noted. By contrast, in healthy controls the alveolar concentrations of the VOCs under study were virtually unrelated to each other. (Fig. 6).

## Discussion

Within this study, we investigated 53 pediatric T1DM patients in parallel to children suffering from chronic kidney disease and healthy controls. PTR-ToF-MS was used for direct analysis of exhaled VOCs. The findings in children with impaired renal function relative to healthy controls have been published recently^36^. Here we focused on the exhalation of ethanol, acetone, isopropanol, dimethylsulfide, isoprene, pentanal, and limonene by pediatric T1DM patients.

These compounds were selected as they have been previously associated to the disturbed glucose homeostasis or reflect a metabolic link to T1DM-related comorbidities, i.e dyslipidimia and oxidative stress.

The most famous volatile metabolite related to an impaired glucose metabolism is acetone and several studies with diabetic patients point to significantly enhanced acetone exhalation in diabetic patients^34,42–45^. To our surprise, this was not the case in our patient cohort and most probably reflects differences in the experimental setting, i.e. sampling of breath in a real-life setting when patients are in a stable metabolic state. In line with this point of view, no association between blood glucose and acetone was detectable. However, our T1DM patients exhaled significantly higher isopropanol concentrations than their healthy peers and isopropanol was associatied with HbA_1c_ as well as with cholesterol and LDL-cholesterol levels in blood. While the exspiration of isopropanol might mirror endogeneous uptake secondary to the use of disinfectant, this will hardly translate into the associations mentioned above. Instead, the reduction of acetone to isopropanol by means of a nicotinamide adenine dinucleotide dependent redox-reaction has to be considered^46^. The formation of isopropanol contributes to acetone metabolism and this pathway may be activated in T1DM patients and thus contributing to acetone elimination and indicating metabolic adaptation.

Isoprene (2-methyl-1,3 butadiene) and limonene (1-Methyl-4-(prop-1-en-2-yl)cyclohex-1-ene) both belong to the family of monoterpenes. Isoprene is supposed to be linked to the biosynthesis of cholesterol and is most probably stored in fat tissue^47,48^. While limonene did not show differences between healthy controls and T1DM patients, isoprene concentrations were elevated in the T1DM group. Previous studies describe similar isoprene concentrations in healthy children and children with T1DM^42^ or even elevated concentrations in adults during hypoglycemic phases^49^. However, the number of participants was low in both cases and no controlled alveolar sampling was applied. As isoprene is known to be largely effected by physiological variations^41,47,50,51^, this may have influenced these results.

Endothelial dysfunction is known to be present in T1DM patients^52–54^ and recent research suggests that endothelial dysfunction may already emerge in diabetic children, even if no atherosclerotic structural changes have been observed yet^38,55^. Increased isoprene concentrations in T1DM patients thus may reflect damages at the cellular level that will translate in altered physiological properties of the endothelium. Furthermore, long-term hyperglycemia might cause vasoconstriction and an inflammatory state with concomitant oxidative stress, which in turn is mirrored by an exhalation of elevated amounts of pentanal. Such structural damage may further influence substance distribution and transport between compartments, leading to alterations in VOC exhalation.

An increase of aldehyde concentrations in T1DM patients, as observed for pentanal in our data, might indicate an elevation of oxidative activity. Oxidative stress is associated with chronic microinflammation and may be to blame for many if not for all of the long-term sequelae seen in diabetic patients^7,56^. However, it is difficult to verify oxidative stress and it has yet to be clarified if elevated pentanal concentrations originate from oxidative stress or an impairment of the antioxidant response.

Dimethylsulfide mainly originates from bacterial activity^57,58^ and may be a breakdown product of methionine^59^. Elevated concentrations in T1DM patients thus may be explained by alterations of the gut microbiome under permanent insulin-deficiency. This might also result in an increased endogeneous production of ethanol, which was elevated in T1DM patients, too^60^. Further, Galassetti *et al*. found rapidly increasing ethanol concentrations in the breath of healthy subjects directly after ingestion of 75 g of glucose, mirroring the increasing blood glucose concentration, However, ethanol returned to baseline quicker than blood glucose. Authors hypothesized that bacterial ethanol production may be supported by systemic hyperglycemia^61^. This explanation might be valid in our study as well, since ethanol concentrations were highest in patients with poor metabolic control (mean HbA_1c_ above 8%). Simic *et al*. noted elevated endogenous ethanol levels in the blood of patients with diabetes mellitus which would be directly reflected in elevated breath concentrations and hence corroborate this hypothesis^62^.

In conclusion, differences in VOC profiles can be observed between healthy controls and T1DM patients, even in children, where long term effects such as co-morbidities have not emerged yet. Those differences were most pronounced in patients with poor metabolic control, i.e. those with a mean HbA_1c_ above 8%.

Monitoring of poor metabolic control as well as early observation of metabolic adaptation through a non-invasive window via breath profiles in T1DM patients may support the discovery of basic metabolic mechanisms and adaptation early in the progress of T1DM.

## Supplementary information

Supplementary information
