# Author Correction: Exhaled volatile substances in children suffering from type 1 diabetes mellitus: results from a cross-sectional study

**DOI:** 10.1038/s41598-020-58619-x

**Published:** 2020-01-29

**Authors:** Phillip Trefz, Juliane Obermeier, Ruth Lehbrink, Jochen K. Schubert, Wolfram Miekisch, Dagmar-Christiane Fischer

**Affiliations:** 1 Department of Anesthesiology and Intensive Care Medicine, Rostock Medical Breath Research Analytics and Technologies (ROMBAT), Rostock University Medical Centre, Rostock, Germany; 2 Department of Pediatrics, Rostock University Medical Centre, Rostock, Germany

Correction to: *Scientific Reports* 10.1038/s41598-019-52165-x, published online 31 October 2019

The Article contains a typographical error in Table 2 for the concentration range of the patient group for DMS.

The correct Table 2 appears below as Table 1.

**Table 1:** Exhaled amounts of selected alveolar VOCs in the study population.

		Ethanol [ppbV]	Acetone [ppbV]	Isopropanol [ppbV]	DMS [ppbV]	Isoprene [ppbV]	Pentanal [ppbV]	Limonen [ppbV]
Controls		82.4^a,b,c^ (20.7–554)	232* (186–306)	784^a,b^ (287–28,963)	10.0^a,b,c^ (0.99–151)	49.6^a,b,c^ (7.44–153)	5.30^a,b,c^ (1.36–36.9)	51.8 (4.81–1,192)
Patients		171^a^ (44.7–1856)	238* (204–256)	1223^a^ (481–15011)	19.6^a^ (4.28–89.6)	112^a^ (8.36–291)	13.5^a^ (6.11–101)	66.7 (13.9–513)
HbA1c< 8%		150^b^ (71.6–1203)	238 (211–256)	924^c^ (481–2509)	20.0^b^ (4.53–89.6)	105^b^ (49.0–291)	10.6^b,d^ (6.11–30.9)	66.7 (14.8–513)
HbA1c> 8%		221^c^ (44.7–1856)	240 (204–255)	1607^b,c^ (686–15011)	17.1^c^ (4.28–44.1)	118^c^ (8.36–265)	16.2^c,d^ (8.27–101)	66.8 (13.9–209)

Data is given as median and range. Superscripts denote significantly different concentrations of the respective analytes between identically labelled groups. DMS, dimethylsulfide.

^a^controls vs patients: p < 0.001 for ethanol, DMS, isoprene, and pentanal, p = 0.002 for isopropanol; ^b^controls vs patients with good metabolic control (HbA_1c_ < 8%): p < 0.001 for ethanol, DMS, isoprene, and pentanal; ^c^controls vs patients with poor metabolic control (HbA_1c_ > 8%): p < 0.001 for ethanol, isopropanol, isoprene, and pentanal, p = 0.03 for DMS; ^d^patiens with poor vs those with good metabolic control: p < 0.001 for isopropanol and p = 0.012 for pentanal.

Additionally, the Article contains an error in Figure 2 whereby the data from three diabetic patients was accidently omitted.

The correct Figure 2 appears below as Figure 1.

**Figure 1:**
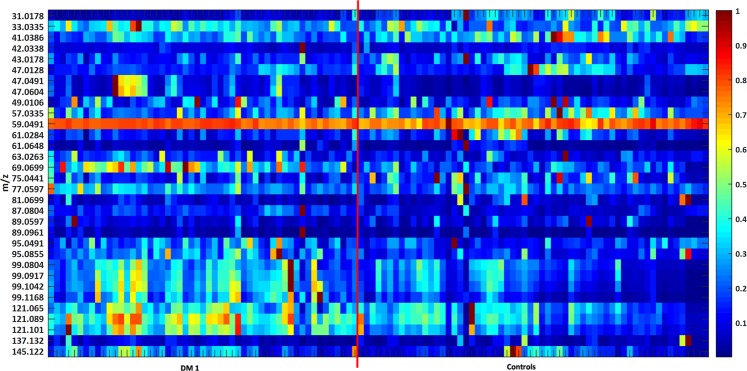
.

