# Supplementary material for: Exhaled volatile substances in children suffering from type 1 diabetes mellitus: results from a cross-sectional study
Source: Sci Rep. 2019 Oct 31;9:15707. doi: 10.1038/s41598-019-52165-x (PMC6823423; doi:10.1038/s41598-019-52165-x)
Supplement: Supplementary file 1 — Supplementary information [file 41598_2019_52165_MOESM1_ESM.pdf]

Exhaled volatile substances in children suffering from type 1 diabetes mellitus: results from a cross-sectional study

Phillip Trefz[1,*], Juliane Obermeier[1], Ruth Lehbrink[2], Jochen K Schubert[1], Wolfram Miekisch[1] and Dagmar-Christiane Fischer[2]

[1]Department of Anesthesiology and Intensive Care Medicine, Rostock Medical Breath Research Analytics and Technologies (ROMBAT), Rostock University Medical Centre, Rostock, Germany, [2]Department of Pediatrics, Rostock University Medical Centre, Rostock, Germany

*Corresponding author

E-Mail: phillip.trefz@uni-rostock.de

Supplementary Information

Supplement 1: Characteristics of patients categorized according to long-term metabolic control.

	HbA1c < 8.0% (14m / 10f)	HbA1c > 8.0% (18m / 11f)
Age [year]	12.21 ± 3.17	12.49 ± 3.18
height [SDS]	-0.17 ± 0.86	0.06 ± 0.88
weight [SDS]	-0.23 ± 0.77*	0.43 ± 0.75
BMI [SDS]	0.11 ± 0.70**	0.55 ± 0.64**
BP_{sys} [SDS]	1.13 ± 0.96**	2.28 ± 1.07**
BP_{dias} [SDS]	0.83 ± 0.97*	1.35 ± 1.34**
Duration of Disease [year]	2.83 (0.17 - 10.92)	4.30 (0.25 - 15.25)
Glucose [mmol/l]	8.49 ± 3.94	10.8 ± 5.02
HbA1c [%]		
at time of examination	7.46 ± 0.81**	9.55 ± 1.43**
mean during last year	7.29 ± 0.45**	9.62 ± 1.15**
Cholesterol [mmol/l]	3.95 ± 0.74**	4.76 ± 1.02**
LDL-Cholesterol [mmol/l]	2.03 ± 0.60**	2.73 ± 0.88**
HDL-Cholesterol [mmol/l]	1.68 ± 0.38	1.58 ± 0.29
Triglyceride [mmol/l]	0.78 ± 0.52**	1.47 ± 1.22**
normalized Insulindosage [IE/kg/d]	0.37 (0.19 - 0.97)	0.37 (0.19 - 1.05)

Superscripts denote significant differences between patients and controls (*p<0.05; ** p<0.01).
